# Overcoming data collection challenges in rural settings by community engagement initiatives: reflections from the research team

**DOI:** 10.3389/fpubh.2026.1688663

**Published:** 2026-02-06

**Authors:** Maarthi Raja, PK Latha, Gayathri KG, Akshaya Prem Kumar, Sarada Satyamoorthy Garg, Maroof Khan, Guda Joseph Raju, Chekuri Gangaraju, Laura Downey, Vidhya Venugopal

**Affiliations:** 1 NIHR GHRC NCD-EC, Faculty of Public Health, Department of Environmental Health Engineering, Sri Ramachandra Institute of Higher Education and Research, Chennai, India; 2 Faculty of Public Health, Department of Environmental Health Engineering, Sri Ramachandra Institute of Higher Education and Research, Chennai, India; 3 The George Institute for Global Health, New Delhi, India; 4 Palasa Kidney Research Centre, The George Institute for Global Health, New Delhi, India; 5 The George Institute for Global Health, UNSW Sydney, Sydney, NSW, Australia

**Keywords:** community engagement initiatives (CEI), data collection, field challenges, heat-related illness, rural settings

## Abstract

**Introduction:**

Community Engagement and Involvement (CEI) are increasingly recognized as vital in addressing research challenges in rural areas, especially concerning environmental health and heat exposure. The socio-culturally diverse rural communities of India face extreme heat, necessitating tailored CEI approaches for effective community involvement. This study examines CEI strategies in heat-health research in rural South India, identifying challenges encountered throughout the research process and highlighting CEI's role in overcoming them.

**Methods:**

This community-based study was conducted in 16 villages across Srikakulam and Parvathipuram Manyam districts of Andhra Pradesh in 2024, using CEI principles. A situational assessment of the state Heat Action Plan (HAP) was undertaken through desk reviews, key informant interviews, and HEAT-PROTECT based evaluations at community, workplace, and PHC levels. Primary data were collected in two phases April–July 2024 and August–December 2024 and included household and workplace surveys, assessments of heat-related knowledge and coping strategies, physiological measurements, and environmental heat monitoring.

**Results:**

Trust-building strategies, such as Community Advisory Boards (CABs) and early engagement, proved essential in accessing hard-to-reach populations. Collaborative research with community input ensured cultural sensitivity and relevance. Continuous engagement and rewarding incentives significantly improved participant retention rates, even in longitudinal studies. Community-led initiatives effectively addressed challenges like language barriers and remote data collection, enhancing data quality and long-term sustainability.

**Discussion:**

CEI strategies are instrumental in simplifying heat-health research complexities in rural Andhra Pradesh. By fostering trust and active participation, CEI strengthens research processes and outcomes. Future studies must prioritize these approaches to enhance research relevance and impact, especially for vulnerable rural populations facing extreme heat.

## 1 Introduction

Climate change has intensified heat stress globally, posing significant environmental and public health challenges. The increasing frequency and intensity of heat waves, particularly in low-and-middle-income countries (LMICs), are raising serious concerns about heat-related morbidity and mortality. Vulnerable populations, including the older adult(s), children, outdoor workers, and low-income families, are disproportionately affected by extreme heat, leading to conditions such as heat exhaustion, heat stroke, cardiovascular diseases (1), and psychological distress. Alarmingly, heat-related deaths have risen by 85% among the older adult(s) between 2017 and 2021, compared to 2000–2004. India, in particular, has witnessed recurrent heat waves, resulting in over 8,000 fatalities between 2015 and 2019 (2, 3), underscoring the urgent need for targeted interventions and adaptive measures.

As a possible means of preventing extreme heat, governments have set up Heat Action Plans (HAPs) in many cities to help protect vulnerable populations, including major US cities, such as Philadelphia and Chicago. HAP has been implemented in India too to mitigate the impact of extreme heat, incorporating strategies such as early warning systems, public awareness campaigns, and healthcare preparedness (4). Ahmedabad was the first Indian city to introduce a comprehensive HAP in 2013, which significantly reduced heat-related mortality (5) and also highlighting the need for context-specific strategies to protect rural populations from extreme heat (6). This model has been replicated in other states, including Odisha, Maharashtra, Telangana and Andhra Pradesh, While Andhra Pradesh has a state-level HAP framework in place, its implementation in rural settings remains partial and uneven, with limited documentation and no published community-based research evaluations (CBR) (7) assessing its on-ground effectiveness.

CBR evaluating HAPs globally has emphasized the importance of localized engagement in reducing heat-related health risks, particularly among vulnerable populations (8). In Canada, Bassil and Cole (9) highlighted how participatory planning and inter-sectorial collaboration improved community responsiveness to heat events. In the United States, Petkova et al. (10) and Hess and Ebi (11) emphasized the effectiveness of collaboration between local health departments, non-profits, and residents in enhancing heat preparedness. In Europe, Lemonsu et al. (12) showed how public awareness campaigns and neighbourhood-level social support systems contributed to increased resilience during heatwaves in Paris.

In India, Azhar et al. (5) evaluated South Asia's first HAP in Ahmedabad, demonstrating the value of community outreach, health worker training in reducing mortality. More recently, (13, 14) assessed community readiness in India, Bangladesh, and Pakistan, and highlighted the importance of participatory vulnerability mapping and local knowledge integration. Despite these advancements, no community-based evaluation study has been conducted on the implementation or effectiveness of the HAP in Andhra Pradesh. Challenges such as limited access to local data, lack of institutional coordination, and difficulties in engaging at-risk populations have hindered such efforts and remain underreported in both research and policy discussions.

To bridge the research lacuna, we conducted investigations to determine the current status of existing implemented HAP in rural communities of Srikakulam and Parvathipuram Manyam districts in Andhra Pradesh (AP). It also aimed at assessing the heat profile of the study area, prevalence of heat-related illness (HRI) and physiological heat strain, knowledge level on heat-related illness and heat coping strategies, and its prevention strategies at the community level, Primary Health Centers (PHCs) level, and at the workplace level. CBR is rapidly gaining recognition as an essential tool in addressing complex environmental, health, and social problems (14). Conducting community-based studies presents numerous challenges that can affect the quality and outcomes of research. This paper aims to share a research team's experience in community engagement and involvement (CEI) and its role in overcoming challenges in the present community-based study conducted in Andhra Pradesh, South India. The objective is to reflect on the appropriateness of how CEI was approached, outline the challenges experienced, and provide insights on how the research team overcame many of these challenges. Rather than reporting on health outcomes or intervention impacts, this paper uses a methodological lens to reflect on our team's lived research experiences. It highlights how CEI served not only as a supportive tool but also as a core enabler of ethical and efficient data collection in rural settings a perspective seldom captured in literature on climate-health research.

While the broader study sought to assess heat vulnerability through quantitative data, this particular manuscript focuses on methodological reflections specifically, how CEI approaches helped navigate complex barriers across all stages of the research cycle, from planning to field implementation and follow-up. It thus aims to contribute to the methodological discourse on conducting ethically robust and logistically feasible research in under-resourced, climate-vulnerable rural settings.

## 2 Study site

**Figure 1:**
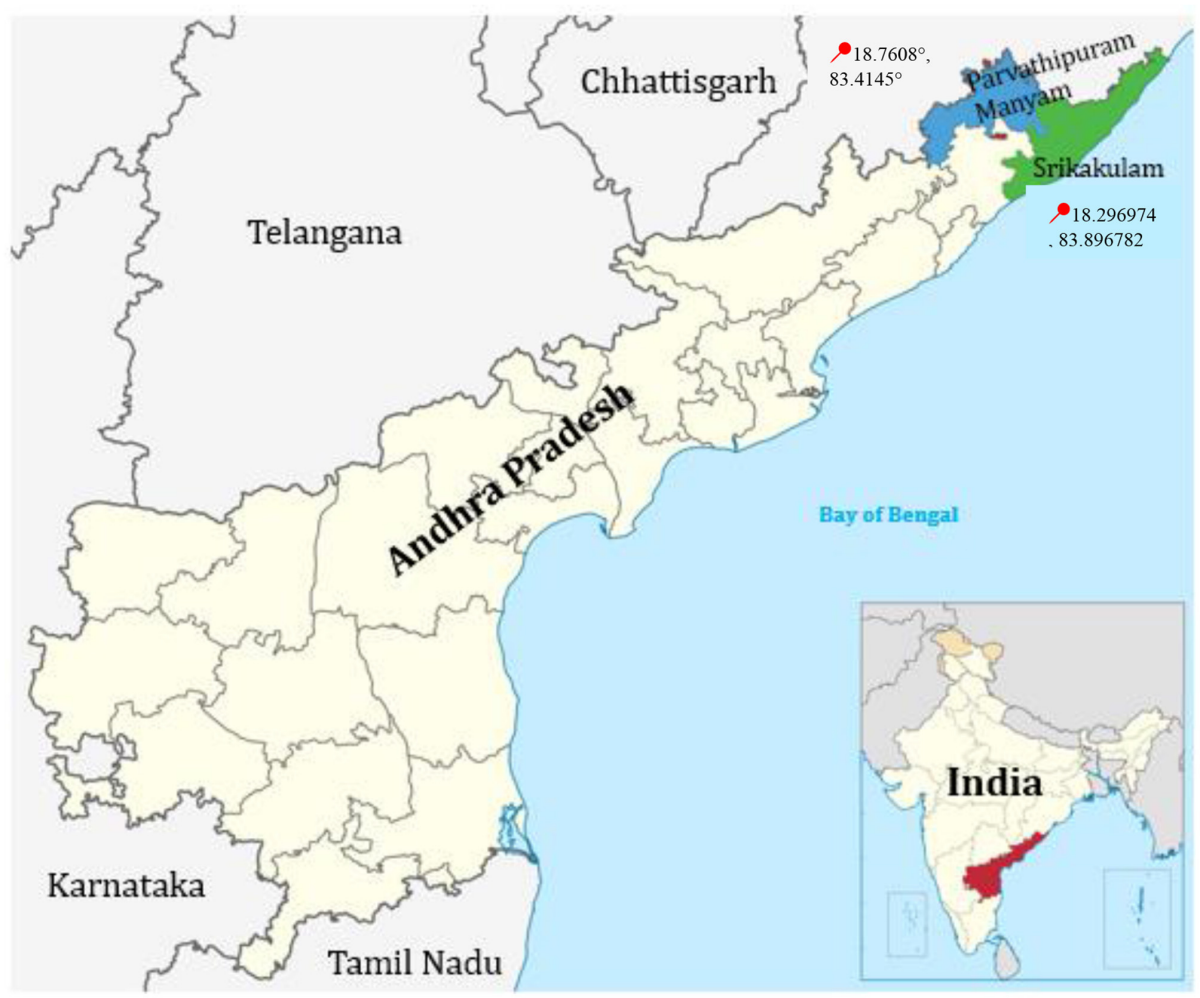
Andhra Pradesh map with latitude and longitude of work place districts.

The investigation was conducted in 16 villages in two districts of Andhra Pradesh, Srikakulam and Parvathipuram Manyam, which are categorized under eight PHCs and their associated workplaces (Figure 1). The villages were selected carefully to ensure comparability and reduce bias while running an intervention trial. Ideally, both locations had a similar demographic profile, socio-economic status, climate conditions, and access to healthcare services.

## 3 Study population

The target population at the community level was the community members aged between 18 and 60 years and who had lived in the same household for more than 6 months. However, individuals with chronic kidney disease (CKD), and those residing in air-conditioned households were excluded. Workers included aged 18–60 years and assessed a range of heat-exposed workplaces, both indoor and outdoor, typical of rural occupational settings in the study districts. These workplaces comprised brick kilns, cashew-processing units, agricultural fields, welding workshops, pottery units, and wood-cutting sites. Only workers who had been employed in the same or similar workplace for at least 6 months were included to ensure consistent exposure history. This selection captured a diverse spectrum of heat exposures, reflecting real-world occupational environments where heat-related illness risks are prevalent in rural Andhra Pradesh. At the PHC level, all the front-line workers, including ASHA workers, health supervisors, ANMs & medical officers were included in the study.

## 4 Study design

This study was conducted as CBR with a dual approach:

- **Preliminary HAP assessment:**

To contextualize the study within the ongoing HAP in Andhra Pradesh, we conducted a situational analysis of existing HAP implementation. This included:

- Desk review of HAP documents at the state and district level.
- Key informant interviews with PHC officials, district health officers, and local administrators.
- The status of the existing HAP was assessed using the HEAT-PROTECT questionnaire, an adapted version of HOTHAPS (15) at three levels: community, workplace, and PHCs. The adapted tool underwent internal review, external expert validation, and pilot testing before deployment. Digital platforms like REDCap (32) and SMART*health* were utilized for the data collection.

- **Primary data collection for heat vulnerability and CEI outcomes:**

The primary focus of this manuscript, and the core of our research, involved CEI-driven data collection to assess heat vulnerability, coping strategies, and physiological heat strain at the community, workplace, and PHC levels. This included

- Household and workplace surveys (brick kilns, cashew-processing units, farms, welding workshops, pottery units, wood-cutting sites) with participants aged 18–60 years who had ≥6 month's exposure in the same or similar setting.
- The awareness level on heat coping strategies were assessed using a modified High Occupational Temperature Health and Productivity Suppression (HOTHAPS) questionnaire through REDCap and SMART*health* digital platforms.
- Physiological measurements (core body temperature, blood pressure, heart rate, urine specific gravity) and heat exposure monitoring (Wet Bulb Globe Temperature (WBGT), heat index) pre- and post-work shifts to determine cross-shift changes in heat exposure and physiological heat strain in workplaces. We also collected urine and blood samples to assess the effects of heat on the workers' renal health.
- Knowledge and awareness assessment using modified HOTHAPS questionnaires via digital platforms.

This approach followed CEI principles, including development of survey tools, training community members as data collectors, and establishing feedback loops to incorporate participant insights.

## 5 Research approach

The research activity involves a situational analysis phase of Implemented Heat Action Plan at Andhra Pradesh, during which data collection occurred at two different time points: pre-during summer assessment (April–July 2024) and post-summer assessments (August–December 2024) in Srikakulam and Parvathipuram Manyam districts. The study also received ethical approval from the Sri Ramachandra Institutional Ethics Committee (IEC/N1/22/NOV/85/113) before the initiation of the study. The research activity involved the following tasks:

- **Stakeholder mapping:** stakeholder mapping in Srikakulam district mapped out major community-level stakeholders, government representatives, health workers, local leaders, self-help groups, and NGOs involved in the implementation of HAP. Their influence and roles were evaluated to inform the effective implementation of the Heat Action Plan.
- **Stakeholder sensitization:** the stakeholders were sensitized to the project goals by conducting in-person meetings and discussions with them.
- **Recruitment of the participants:** the recruitment was conducted through convenience sampling according to the particular inclusion and exclusion criteria laid out in Section 3. Community trust-building measures ensured that willingness to participate did not skew recruitment. CEI strategies were crucial in minimizing selection bias and enhancing participation from the most heat-vulnerable groups.
- **Heat vulnerable village:** a portable device was employed to record WBGT, ambient temperature, and humidity to determine the study area's heat index (HI) trend. Villages were assessed for heat vulnerability on the basis of environmental parameters (temperature, humidity, power outages, and ventilation) and socioeconomic parameters (income, dwelling, green spaces, access to healthcare, and awareness of heat illness). A composite vulnerability score was calculated to identify the village with the most at-risk population.

We followed a **Community-Based Participatory Research (CBPR)** strategy that significantly improved stakeholder collaboration and inclusivity. In recent literature demonstrates that community-based/participatory research (CBR/CBPR) approaches are being increasingly applied in climate and health research to ensure interventions are locally relevant, ethically grounded, and effective (16–18). This strategy involved partnership with the community throughout the research process—from planning and design to data collection, interpretation, and dissemination. Our strategy began with meticulous planning and preparation, establishing well-defined goals and identifying significant stakeholders. The Key principles embraced were:

- **Early engagement** of local stakeholders and leaders to establish trust and secure buy-in.
- **Culturally sensitive communication**, in local languages and familiar formats (e.g., transect walk with Community Leader, community meetings).
- **Training and engaging community members** as data collectors to produce local relevance and increase ownership.
- **Feedback loops** to report findings to the community and incorporate their observations into furthering intervention for the future.
- **Respect and acknowledgment of local knowledge**, ensuring study is consistent with the community's priorities and values.

## 6 Stakeholders mapping and engagement

Stakeholder mapping entailed the identification, analysis, and prioritization of persons and groups affected based on their impact, interest, and relevance to the study for focused engagement guidance strategies. Table 1 below identifies the main stakeholders involved in the study. Regular stakeholder engagement was conducted, which included regular consultations with local leaders, health workers, and community groups to guarantee their active participation in the research activity (see Table 1).

**Table 1:** Stakeholders engaged in the study and their respective roles.

**S. No**	**Stakeholders**	**Roles**	**Engagement benefit**
1	District collector	Highest-ranking official at the district level	Granted government permissions, aligned study with district priorities, provided legitimacy and promoted cooperation from local authorities.
2	District Medical and Health Officer (DMHO)	Oversees public health programs in the district level	Ensured integration with current health plans, provided district-level health data, and coordinated research team with health personnel.
3	Primary Health Centre (PHC) staff	Fieldwork facilitators and community intermediaries	Assisted in securing permissions, communicated study purpose to the public, collected data, and supported follow-ups.
4	Local NGOs	Community engagement and support	Helped access remote areas, educated communities on heat risks, facilitated data collection, and identified key stakeholders for prior approvals.
5	Village health workers (ASHAs, ANMs, etc.)	Primary community links in rural areas	Facilitated participant recruitment, collected health data, and educated communities on heat-related illnesses.
6	Village president and local leaders	Community trust builders and facilitators	Provided access to village resources, ensured community engagement, and facilitated availability of study spaces.
7	Workplace owners	Key stakeholders for worker engagement	Provided workplace access, minimized workflow disruptions, supported health monitoring, and promoted heat-safety measures.
8	Study participants (Individuals & Workers)	Data providers and community representatives	Shared insights through surveys and interviews, contributed to understanding heat stress coping mechanisms and workplace vulnerabilities.
9	Project staff	Study management and coordination	Managed project implementation, ensured ethical oversight, instructed field investigators, and maintained communication with key stakeholders.
10	Field investigators	Community liaisons and data collectors	Conducted surveys and interviews, ensured accurate data collection, built participant trust, and facilitated study retention.

## 7 Barriers and role of CEI in teasing out the barriers

### 7.1 Pre-field challenges

a. **Permission to conduct the study:** language barriers and scheduling challenges at the community level significantly impeded obtaining permission from the village head. Management's apprehensions regarding disruptions to their work schedules posed a challenge in obtaining data collection permissions for heat-exposed individuals in the workplace. Management was also concerned about regulatory oversight and privacy concerns in the workplace. Additionally, inefficient workflows and conflicting priorities made it challenging to access public health data at the primary healthcare level.

**CEI approaches:** To overcome delays and barriers related to permissions, workplace concerns, and PHC data access, a tailored CEI approach was adopted that emphasized early stakeholder mapping and Building trust with local gatekeepers. The community advisory boards comprised village leaders, frontline health workers (ASHAs, Anganwadi workers), PHC medical officers, local NGO representatives, and community members from diverse occupational groups.

Village leaders initially dominated discussions, potentially silencing marginalized voices. We addressed this by conducting separate focus groups with different stakeholder categories before joint meetings and rotating meeting venues to neutral spaces. For example, ASHA workers hesitated to share service delivery challenges in the presence of PHC officials, so we held separate ASHA-only sessions where they candidly discussed resource constraints. These strategies helped balance power dynamics and ensured more equitable participation (16) and flexible schedules were co-developed with input from village leaders and workers to reduce disruptions and improve participation. Formal prior agreements with PHCs and workplaces by our partnering institution, which has already established trust with the relevant stakeholders, addressed concerns around privacy and oversight, while transparent communication and regular feedback loops helped sustain trust. Also, the trust built with the District Medical Officer and healthcare personnel by the partnering institution promoted strong collaboration. Local staff were involved in project activities to enhance ownership and minimize resistance, paving the way for smoother implementation.

b. **Sensitization of the importance of the study:** language and cultural considerations were integral to the research process. Several stakeholders including village leaders, healthcare workers, workplace owners, and community participants initially faced challenges in comprehending the study's objectives and procedures due to linguistic and conceptual barriers.

To address this:

• Technical terms such as “heat stress” were translated using culturally meaningful equivalents.

• Questions about water intake were adapted from litres to traditional vessel measurements.

• Likert scales were simplified for participants with limited formal education.

Additionally, occupational classifications were revised to capture seasonal and livelihood-specific variations. Gender norms influencing women's participation in mixed-group discussions were carefully considered, and time-related questions were reframed according to activity-based daily routines rather than clock hours. Steps were also taken to minimize translation inaccuracies that could lead to misinterpretation of key study concepts.

**CEI Approaches:** to address linguistic and cultural challenges, we implemented community engagement strategies throughout the research process. ASHAs and Anganwadi workers pre-tested tools and provided feedback for iterative refinement, while local volunteers developed culturally appropriate translations and female health workers facilitated gender-segregated discussions. Native Telugu-speaking staff members participated in stakeholder meetings to ensure effective communication. Fieldwork was conducted by local investigators who were culturally knowledgeable, fluent in the native language, and trusted within their communities. This approach improved instrument validity, participant comfort, and data quality while bridging communication gaps and building trust. Early and sustained engagement fostered strong relationships between researchers and participants, significantly enhancing information exchange and community participation throughout the study.

c. **Building Trust with Significant Stakeholders:** building trust with stakeholders such as district authorities, village chiefs, business owners, health officials, and research participants proved to be a considerable challenge. Coordinating meetings with district-level authorities, including the collector and the medical officer, was difficult due to their busy schedules and geographical constraints. Concerns about the study's purpose and the potential benefits for their communities further complicate the process of obtaining necessary permissions. Additionally, many stakeholders, including participants, initially hesitated to engage with researchers outside their local communities, leading to further delays in establishing the trust needed to proceed with our research.

**CEI approaches:** we implemented several effective strategies to build trust among stakeholders and participants. Engaging in initial and ongoing dialogues with district authorities and local leaders through local NGOs ensured transparent communication about the study's objectives and the benefits it could bring to the community. Involving these groups in the planning process allowed us to address their concerns and create confidence in our goals. Community advisory boards facilitated conversations between researchers and community members, helping to clarify misunderstandings and encouraging valuable feedback. Stakeholders participated in workshop on potential interventions for the area, which empowered them and provided a sense of ownership over the research process. These approaches fostered trust, improved interactions, and supported effective implementation of the research.

d. **Access to interior rural villages:** limited connectivity significantly restricted our access to participant populations in the remote rural villages targeted for this study. The research team faced significant hurdles in reaching remote study locations, primarily because of poor road conditions, including unpaved and unmarked routes. The team had to invest considerable time in understanding the unique landscape, culture, and logistical challenges. A lack of clear directions and reliable information about the terrain made planning our study visits challenging and delayed our fieldwork schedule.

**CEI Approaches:** We used a collaborative partnership approach approaches (collaborating with local non-governmental organizations, engaging community leaders, and consulting local advisory groups) to leverage local resources and expertise to reach remote areas. Our first collaboration with a local NGO with a strong presence in the area, and who knew the terrain, helped us navigate effectively and engage community members. Working alongside the village leaders and local advisory groups was followed by the team to efficiently ensure that field visits had adequate schedules, optimal routes, and appropriate transportation methods to save much time and resources for the project. Involving the communities and co-opting them in the initiatives also motivated local stakeholders to actively engage in the project actively, thereby opening up previously inaccessible areas to a much greater extent (see Table 2).

**Table 2:** Summary of the key challenges faced before the data collection and the CEI approach.

**Challenges**	**Description**	**CEI approach**
Obtaining permission for conducting the study.	Language and scheduling barriers delayed permission from village heads.	• **Stakeholder engagement:** early mapping, trust-building with gatekeepers, and community advisory boards formed. • **Collaborative planning:** flexible schedules co-developed with input from village leaders and workers • **Institutional support**: pre-existing MoUs with PHCs/workplaces eased privacy and oversight concerns. • **Sustained trust and ownership:** transparent communication, DMO collaboration, and local staff involvement ensured buy-in.
Sensitisation of the importance of the study.	Misinterpretations hindered communication and trust.	• **Linguistically inclusive engagement:** employed native Telugu-speaking staff during stakeholder meetings and discussions. • **Early and sustained engagement:** initiated and sustained long-term community interaction
Building trust with the stakeholders.	Discomfort with non-native researchers hindered trust and understanding among stakeholders.	• **Culturally sensitive staffing:** recruited native Telugu-speaking staff and culturally knowledgeable local investigators. • **Early and inclusive engagement:** we engaged community members and stakeholders from the start, which built trust, improved clarity, and strengthened community relationships. • **Community advisory boards**: enabled open communication and feedback. Involving stakeholders in developing interventions promoted ownership and transparency.
Access to Interior study location.	Poor road conditions, lack of clear routes, and unfamiliar terrain delayed field visits and planning.	• **Engagement with local leaders:** worked with village leaders and advisory groups, which optimized field schedules, routes, and transport, saving time and resources • **Community involvement:** co-opted communities in planning and implementation

### 7.2 During field challenges

a. **Participant recruitment and informed consent**: recruiting research volunteers presented several challenges. Many respondents expressed concerns that participating in the research would disrupt their schedules and consume too much time. The unfamiliarity of our study team exacerbated their fear and made them reluctant to share personal information. Securing informed consent was difficult because some participants felt that working in the heat was unavoidable, causing them to question the significance of their involvement in the study. This initial skepticism hindered the development of rapport and team engagement. Additionally, language barriers complicated communication, as most participants were not fluent in English. Participants experienced discomfort when speaking and occasionally responding in a language they were not proficient in. Consequently, articulating the study's objective and responding to their concerns proved challenging.

**CEI Approaches:** we implemented a range of effective strategies to actively engage participants in our study. To start, we consistently involved trusted community leaders and local organizations, fostering a strong sense of trust and clearly articulating the benefits of the research. Once rapport was established, we proactively approached potential participants in their workplaces and within the community, thoroughly explaining the research and addressing any concerns they might have had. Our dedicated field investigators worked closely with local healthcare professionals and village leaders, helping to alleviate participants' worries and reinforce their understanding. This collaboration not only conveyed the study's objectives but also demonstrated robust community support, significantly enhancing participant engagement.

To ensure all participants could make informed decisions, we provided comprehensive information in the local language. For those who struggled to understand the permission form, community members took the time to explain the key details, guaranteeing that everyone had a clear and thorough understanding of what participation entailed. By fostering transparency and community involvement, we aimed to create an empowering environment for all participants.

b. **Field data collection:** field data gathering was particularly challenging due to language barriers encountered while administering questionnaires, collecting biological samples, and taking anthropometric measurements from participants. Most participants only spoke the local dialect, which made it difficult to explain the questionnaires and address their questions. Even field investigators who were locals and were fluent in the native language sometimes became confused, leading to misunderstandings about the importance of specific data needed for analysis.

In addition, there was a difference in the reading abilities of all participants leading to the fact that the clarification period of questionnaires was even longer. Misinterpretations of questions that led to the understanding of the exact meanings of the questions were faced by both participants and investigators. We were able to identify several critical questions that could have caused delays in long-time interviewing, thus lengthening the time taken to interview the participants. We successfully obtained biological samples and anthropometric measurements from most participants. However, some refused to provide their samples due to concerns about malpractice.

**CEI Approaches:** we aimed our CEI methods to bridge the gap between researchers and the community. Making the participants understand the study's purpose and its value to the community was vital in garnering their cooperation and adoption of the intervention. We achieved this with the help of community advisory boards and local leaders who addressed initial concerns and ensured participants felt comfortable with the research team. Continuous engagement and regular interactions with the community from the start of the project were key in building trust, which proved essential in managing challenges during the fieldwork. We worked collaboratively with the community to refine the survey tools, adapting the wording and format based on community feedback to improve linguistic clarity and cultural relevance. This made it easier for them to read since they had varied reading levels, and the overall process was streamlined and more representative. This not only improved the clarity of the survey for participants but also made the participants feel engaged in the process, which meant that our study was more effective in data collection efficiency.

c. **Results distribution from monitoring:** the distribution of test results presented significant challenges, as many study participants were absent upon our return, mainly due to work commitments or being away from their locations. Although tracking all participants was difficult, we were able to successfully monitor the majority of those who were absent. We did, however, fail to inform some individuals about the normal ranges for the monitored health indicators. This oversight caused ambiguity and concern, as the initial assessment did not classify the results as either standard or alarming. Participants struggled to understand the implications of their results, whether they were normal or concerning, which ultimately affected their willingness to participate in intervention studies.

**CEI Approaches:** to tackle this challenge, we implemented proactive engagement strategies with local community leaders to ensure the effective delivery of results to participants. In instances where individuals were unavailable, we collaborated with local leaders and health workers to help distribute results. Additionally, we made multiple visits to households alongside community leaders to ensure thorough follow-up and communication. Every participant who had relocated or was working received their data. We also provided clear interpretations of the typical health ranges associated with the results. Follow-up meetings were held in which health workers explained the significance of the test results, established normal ranges, and outlined appropriate actions for results that exceeded these limits. This process enabled most participants to better understand their health data, alleviating concerns and misconceptions. Additionally, local leaders shared stories of past successful health interventions, which enhanced participants' willingness to engage in the study (see Table 3).

**Table 3:** Summary of the key challenges faced during the data collection and the CEI approach.

**Challenges**	**Description**	**CEI approach**
Participant Recruitment	• Concerns about time and disruption. • Reluctance due to unfamiliar researchers. • Skepticism regarding the relevance of the study. • Language barriers and discomfort with English. • Difficulty in explaining study goals and securing consent.	• **Engagement with local stakeholders**: involved trusted community leaders and local organizations. • **Proactive and personalized engagement:** we approached participants in their workplaces and communities, explained the study purpose, and addressed concerns. • **Collaborative fieldwork:** field investigators worked with local healthcare professionals and village leaders
Field data collection	• Language barriers during data collection. • Varied reading abilities among participants. • Misunderstood questions. • Concerns over providing biological samples.	• **Participant empowerment:** involved participants in shaping the research process. • **Community-researcher bridging:** used CABs and local leaders to address concerns and promote comfort with the research team. • **Collaborative planning:** adapted wording and format of tools based on community feedback and understanding levels. • **Built-trust:** ensured that the health checkup results were distributed in person immediately.
Report distribution	• Difficulty tracking participants. • Confusion over test result interpretation. • Concerns due to lack of explanation about normal health ranges.	• Proactive community engagement, household follow-ups, clear health communication, use of local narratives.

### 7.3 Post-field activity challenges

a. **Cohort retention for future visits:** maintaining participant retention for follow-up visits across the seasons was a significant challenge. Many participants, especially seasonal workers, changed jobs, which complicated our efforts to keep them motivated to participate throughout the study. Additionally, some participants expressed concerns that the time commitment required might interfere with their regular routines or work schedules. As a result, ensuring consistent participation and collecting data from the same cohort over multiple seasons proved difficult.

**CEI approaches**: to promote participation, we organized neighbourhood gatherings during the off-season. We worked together with field-level local health workers, who were the first link between the communities and the health system, and community leaders to educate and activate participants on the importance of continuous participation throughout the year. A flexible follow-up system was developed to allow participants who relocated or changed jobs, which included phone tracking and rescheduling visits. We developed a follow-up plan that was convenient for the participants, keeping the disruptions in their daily life to a minimum. We ensured that their time and efforts were compensated for maintaining their commitment. These strategies ensured retention and ensured that data could be collected uniformly across all three seasons, hence preserving the integrity of the study.

b. **Secondary data collection:** from the beginning of the project, secondary data collection posed challenges and continued throughout the research. Collecting retrospective health records, and environmental data, from the regional and local government offices or healthcare facilities was difficult to obtain often due to bureaucratic delays and complications in data management. We could not easily get the necessary clearances from the key district-level authorities, including the district collector, the District Medical and Health Officer (DMHO), and the state public health departments. Getting the clearances needed more time and layers of authorization, which really disrupted our efforts to collect some of the necessary secondary data required for the study.

**CEI approaches**: to address this challenge, we initiated discussions with pertinent district authorities, including the collector, DMHO, and other officers. We conducted formal meetings and gave presentations, emphasizing the significance of the study, the necessity of secondary data collection, and the potential benefits for the community. We also approached the state-level authorities with a request for secondary data and assured the diligent use of the data provided. Maintaining continuous communication was critical for obtaining the necessary approvals. We collaborated with local healthcare providers and community advisory boards that had already established trust with these institutions. After significant effort, we received some environmental and health data from state-level agencies. Community connections made it easier to get data and ensured that valid secondary data was gathered, improving the research quality (see Table 4).

**Table 4:** Summary of the key challenges faced during post-data collection and the CEI approach.

**Challenges**	**Description**	**CEI approach**
**Cohort retention for future visits**	• Seasonal migration and time constraints disrupted follow-up and data collection from the same participants across all seasons.	• **Community activation and flexibility**: organized off-season neighbourhood gatherings. • Educated on long-term participation value.
**Secondary data collection**	• Bureaucratic delays and difficulty in accessing government health and environmental data from district and state authorities.	• **Stakeholder engagement and advocacy:** Conducted meetings and presentations with district-level officials (e.g., Collector, DMHO). • **State-level coordination and persistence:** Approached state health authorities for clearance. • Maintained ongoing follow-up and committed to ethical data usage.

## 8 Discussion

Community-based research often poses various challenges methodological, ethical, cultural, and logistical—for example, problems with participant recruitment, cultural sensitivity, and management of stakeholders hindering the effectiveness and impact of the research. Community-based research presents just as many unique opportunities for meaningful engagement and outcomes locally relevant. Even though there is an issue with ethics and practicality, these studies provide essential information regarding what the community needs, and they support working together to find solutions that may not be possible with traditional research methods. This study contributes to the growing body of evidence on operationalizing CEI in heat-health research in rural, resource-constrained settings. By balancing ethical priorities and pragmatics, the CEI approaches outlined herein were not just central to developing trust within the community and encouraging participation, but also to navigating socio-cultural dynamics that commonly serve as hindrances to public health research. Notably, our work in Andhra Pradesh reaffirms the value of embedding CEI across all research phases from early planning to post-field feedback thus enhancing both ethical rigour and contextual relevance. The literature consistently highlights CEI as a cornerstone of ethical and effective health research, especially in settings marked by historical inequities and power differentials (19, 20). Our own experience confirms these findings, showing how initial consultations with local stakeholders and ongoing dialogue with community gatekeepers facilitated the co-production of research processes that were respectful, inclusive, and responsive to local needs. Engagement in this way not only facilitated rapport but also helped to avoid potential misperceptions about the purpose of the study the usual risk in under-researched settings where experience of formal research is non-existent or minimal (21).

One of the strengths of our strategy was prioritizing contextualization of CEI practices to the occupational and cultural realities of the community. For example, the use of local health workers and NGO staff as “cultural brokers” facilitated filling in gaps in communication and allowed us to negotiate hierarchies in the health system and the community. This resonates with the views of George et al. (22), who posit that effective CEI tends to rely on whether the research team can align engagement mechanisms with the local knowledge systems and power arrangements. Our results further align with the ethical standards outlined by Emanuel et al. (23), specifically those of partnership and benefit to the community. The informal post-study debriefs and feedback sessions we conducted evidence a commitment to reciprocity, recognized in literature as an important but frequently under-delivered element of community engagement (24). Our systematic effort to return findings and recognize community investment, even non-materially (e.g., by recognition or dissemination), reinforced participant sense of ownership and dignity, and set the platform for future partnership.

Nonetheless, our research also faced some of the enduring tensions well-cited in CEI literature such as striking a balance between community participation and scientific goals, and navigating expectations regarding benefits to participation (25, 26). For example, although communities appreciated having the researchers there and were interested in ongoing participation, the failure to provide quick fixes to mitigate their occupational heat stress often resulted in frustration. This highlights a more general issue in public health research, where participation can create expectations beyond what the project is capable of fulfilling (27, 28). Clear communication around the boundaries and limitations of research was essential in the management of these expectations and in trust-building. The second limitation is the resource intensiveness of effective CEI. Like other field-based research (29), our researchers were faced with time and logistical limitations, especially with the running of iterative community forums and adapting our materials to be linguistically and culturally appropriate. However, these investments were important: they improved the quality of our data and promoted richer understanding of rural home-based care workers' lived realities, a group that continues to be marginalized in climate and occupational health literature.

Importantly, the collaborative and participatory nature of our CEI also had a reflexive effect on the research team. It prompted critical reflections on power dynamics, researcher positionality, and the need to continuously negotiate the boundaries of participation and representation (30). These reflections were not incidental, but integral to developing a respectful, ethical, and responsive research process. Looking ahead, the integration of CEI in community-based climate adaptation research must move beyond instrumental motivations to a more transformative paradigm. This includes recognising community members not merely as participants or informants, but as co-producers of knowledge, capable of shaping research agendas and advocating for structural change (31). Institutional and funding mechanisms must support this vision by allocating adequate time, resources, and training to foster long-term partnerships.

## 9 Strengths

This article discuss the CEI strategies that are important for dealing with challenges that have arisen within community-based studies, particularly those influenced by heat. Effective engagement practices involve setting up community advisory boards to facilitate early and ongoing engagement, in addition to implementing collaborative processes. Focusing on a scenario pertinent to rural South India could usefully highlight under-resourced and hard-to-reach communities that are unique to these contexts. This level of detail makes the results more relevant and usable in similar rural or remote settings. It also gives researchers working in other developing regions useful information. It is crucial to employ culturally sensitive methods to collect data and recruit participants to ensure that all ethical best practices are upheld, particularly in obtaining informed consent and retaining participants. Engaging local stakeholders or trusted community leaders can further enhance participant involvement and foster long-term commitment to the study.

## 10 Limitations

The study was specific to Andhra Pradesh, and the results are not generalizable to other regions because of the differences in language, culture, and governance. Caution needs to be taken when applying these strategies elsewhere. For long-term community engagement in studies, more importance needs to be given to CEI. Unaddressed, ongoing incentives for the community may reduce sustained interest. Such community-led initiatives and development of data tools have resource demands and require strong engagement, which might be challenging for limited funding projects to apply in an effective manner.

## 11 Community-based research: insights for future planning

Our experiences offer key recommendations for future community-based climate health research. Flexible timelines are essential for trust-building, tool development, and stakeholder engagement. Adequate human resources—including trained field staff, translators, and local facilitators—are critical for navigating linguistic and cultural contexts. Budgets must realistically account for community engagement, translation, digital platforms, and rural logistics. These resource allocations are not overhead but essential investments that ensure ethical engagement and improve data quality and research applicability.

## 12 Conclusion

Community Engagement Involvement (CEI) play a great role in heat intervention research, particularly in rural areas, where cultural and logistical problems are at hand for the field workers. Different strategies can be used to engage the community effectively and build trust to allow for effective research progression. These are community advisory boards, ensuring early and sustained involvement, and promotion of community-led projects. This could facilitate the approach toward finding participants, getting consent, and acquiring data. More importantly, this strategy can ensure keeping participants around for long periods in community-based long-term studies.

In a country like India which has a huge rural population, heat vulnerability is expected to be diverse alongside entrenched social and cultural norms making it vital to employ a customized approach to community research and development. CEI acts as a conduit between researchers and communities, aligning study designs and interventions with the voices and needs of vulnerable populations. Engaging with grassroots health workers, key community leaders, and community members is essential for making heat-health interventions both acceptable and adoptable. By integrating community engagement initiatives into heat research, we can develop a more solution-oriented framework that enhances the relevance and impact of previous studies. Prioritizing community engagement ensures that the experiences and insights of local populations guide the creation of effective heat adaptation strategies that are contextually appropriate. This empowering approach aims to strengthen community resilience against the growing challenges posed by rising temperatures in our changing climate.

## Data Availability

The original contributions presented in the study are included in the article/supplementary material, further inquiries can be directed to the corresponding author.
